# Ketones And Cognition: Insights From A Reverse-Translation Approach In Alzheimer Disease Mice

**DOI:** 10.1093/geroni/igaf122.3183

**Published:** 2025-12-31

**Authors:** Karl Fernandes, Paule M’Bra

**Affiliations:** Université de Sherbrooke, Sherbrooke, Quebec, Canada; Université de Montréal, Montreal, Quebec, Canada

## Abstract

Lifestyle factors are estimated to account for 40% of the risk of developing dementia. Despite this, trials of lifestyle-based interventions, including ketogenic dietary interventions, have shown mixed results in delaying dementia and evidence of both responder- and non-responder participants. In order to understand and eventually optimize ketogenic interventions for dementia, we are using a “reverse-translation” approach in which ketogenic strategies that show promise in humans are modeled in transgenic mouse models of Alzheimer’s disease (AD) in order to more clearly understand their mechanisms of action. Behavioral, anatomical and transcriptomic analyses in AD mouse models confirmed that two distinct ketogenic interventions (dietary enrichment with medium chain triglycerides and a high fat/low carb diet) both improved hippocampal-dependent learning and memory and modulated hippocampal neuronal structure and gene expression. Unexpectedly, despite their similar effects on brain function, these two ketogenic interventions showed markedly different effects on circulating ketone levels, suggesting underlying mechanisms that are independent of ketones. Indeed, metabolic and RNA sequencing experiments identified striking, diet-specific effects on multiple peripheral pathophysiological features of AD, including on glucose homeostasis, liver structure-function and the gut microbiome. These findings have important implications for the design of ketogenic and combinatorial lifestyle-based strategies for dementia.

